# Growth pattern from birth to adulthood in African pygmies of known age

**DOI:** 10.1038/ncomms8672

**Published:** 2015-07-28

**Authors:** Fernando V. Ramirez Rozzi, Yves Koudou, Alain Froment, Yves Le Bouc, Jérémie Botton

**Affiliations:** 1 UPR 2147 CNRS, 44 rue de l'Amiral Mouchez, 75014 Paris, France; 2 INSERM U1018, Center for Research in Epidemiology and Population Health and UMRS 1018, University of Paris-Sud, 16 avenue Paul Vaillant Couturier, 94807 Villejuif cedex, France; 3 Collections d'Anthropologie, Musée de l'Homme, MNHN, 75116 Paris, France; 4 Centre de Recherche St-Antoine UMRS-938; Inserm; UPMC-Paris VI; Hôpital Armand Trousseau-APHP, 26 Avenue du Docteur Arnold Netter, 75012, Paris, France; 5 INSERM, UMR1153 Epidemiology and Biostatistics Sorbonne Paris Cité Center (CRESS), Team ‘Early Origin of the Child's Health and Development' (ORCHAD) and University Paris-Sud, Faculty of Pharmacy, 91400 Orsay, France

## Abstract

The African pygmy phenotype stems from genetic foundations and is considered to be the product of a disturbance in the growth hormone–insulin-like growth factor (GH–IGF) axis. However, when and how the pygmy phenotype is acquired during growth remains unknown. Here we describe growth patterns in Baka pygmies based on two longitudinal studies of individuals of known age, from the time of birth to the age of 25 years. Body size at birth among the Baka is within standard limits, but their growth rate slows significantly during the first two years of life. It then more or less follows the standard pattern, with a growth spurt at adolescence. Their life history variables do not allow the Baka to be distinguished from other populations. Therefore, the pygmy phenotype in the Baka is the result of a change in growth that occurs during infancy, which differentiates them from East African pygmies revealing convergent evolution.

## Abstract (web summary)

The African pygmies are known for their short stature, yet it is unclear when and how this phenotype is acquired during growth. Here the authors show that the pygmies' small stature results primarily from slow growth during infancy.

## Graphical abstract

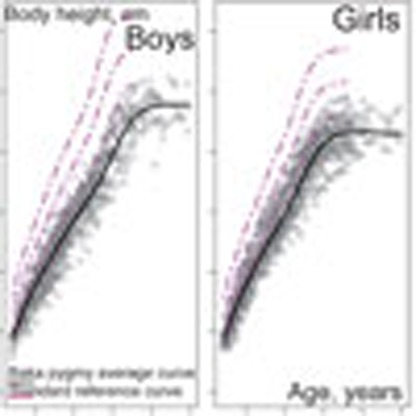

Short stature in human populations is of particular interest to geneticists and evolutionary and cultural anthropologists, as well as to physicians and sociologists. Dwarfism is not restricted to humans: it is part of the so-called ‘island rule', a tendency in vertebrates towards gigantism in small species and dwarfism in large species^1^. Dwarfism is in fact an endemic phenomenon observed in several orders of mammals inhabiting islands, which is probably due to the lack of predation pressure and exposure to limited resources^2^. The ‘island rule' does not only account for these phenomena in geographic islands as it is also observed in ‘environmental islands', where patches of a particular environment are located within and surrounded by other very different environments^3^. Dwarf populations are considered to be the descendants of groups inhabiting the mainland and are regarded as particular sub-species or distinct species.

Populations of short average stature, such as African pygmies, show that growth patterns vary among modern humans, although the mechanisms responsible and their impact on the evolution of modern humans are not well understood. Human populations with short stature exist on all continents. The term ‘pygmies' has been widely used for all of them but with no biological foundation. Since genetic studies have suggested that African pygmies share a common ancestor, restricting the word ‘pygmies' to African populations provides a biological foundation for the term. Furthermore, although other groups of short average stature live in different environments^4^,^5^, restricting the use of ‘Pygmies' to groups who share particular socioeconomic and cultural behaviour patterns and inhabit a similar environment provides a basis for investigations into their anatomical adaptation.

African pygmies live in equatorial rain forests and grow to an average adult stature of <155 cm^6^. They share an economy based on hunting and gathering and a complex socioeconomic relationship with their farming neighbours. Moreover, in each case pygmy peoples are identified by their culture and behaviour as pygmies by farming neighbours, who in turn identify themselves as non-pygmies and are recognized as non-pygmies by pygmies, as well as by other non-pygmies^7^,^8^. Pygmy populations are distributed across equatorial Africa in two main clusters. One is in East Africa (Ruanda, Uganda and Eastern DRC) and comprises (following Schebesta^9^, Gusinde^10^ and many others^11^,^12^ nomenclature) the Aka, Sua, Efe groups (also frequently called ‘Mbuti') and the Batwa. The other cluster, in West Africa (Cameroon, Central Africa Republic, Congo, Gabon and Western DRC), includes the Kola, Bongo, Koya, Aka, Baka and Twa. Pygmies share a common ancestor and splitted from Bantu-speaking populations at around 60,000 years BP^13^; the split into an eastern and a western cluster would have taken place later than 20,000 years BP^13^. Substantial admixtures between pygmies and non-pygmies have occurred in the last ∼1,000 years^14^. Even today, the main barriers to admixture are the cultural and behavioural differences between these two groups^7^. The pygmy phenotype itself is usually interpreted as an adaptation to life in equatorial rain forests^5^. Studies of genetic introgression have shown that the pygmy phenotype stems from genetic foundations^15^, perhaps involving a deficiency in the GH–IGF axis^16^. Although some particular SNPs in genes associated with stature have been identified in pygmies^17^,^18^, the exact genetic foundation remains elusive^19^.

Few studies have suggested any explanation to account for when and how the pygmy phenotype is achieved during individual growth. Merimee^20^, based on blood analyses of Aka pygmies from the Central African Republic, has suggested that the short stature of pygmies could be accounted for by the absence of an adolescent growth spurt because, during this period of growth, pygmies have a low content of IGF1 (pituitary GH effector and hormonal stimulator of cell growth, proliferation and differentiation, particularly in the growth plate). Since the average rate of height gain during this spurt is around 9 cm year in boys (total spurt around 25 cm) and 7 cm per year in girls (total spurt around 20 cm) in most human populations^21^, the absence of this spurt could easily explain the short stature of pygmies. Migliano *et al.*^22^, studying anthropometric measurements and life history variables in populations with a short stature from the Philippines (Aeta), have suggested that their life history variables develop at an early stage in life and that the short stature of these groups is due to relatively earlier cessation of growth. Therefore, according to Merimee^20^, the pygmy phenotype is acquired from adolescence whereas Migliano *et al.*^22^ suggest that rather than a result of environmental adaptation, their short stature may instead be a by-product of an early onset of reproduction, itself a consequence of high mortality rates.

Among primates, humans are distinctive in their very particular growth pattern in absolute and relative terms, which defines what is called the ‘human life-cycle'. Growth in humans is rapid during the first 2 or 3 years of life. A period of linear growth follows, and then a peak in the growth rate during puberty. Human growth is characterized by many aspects that have been widely discussed in previous studies (for example, presence of a childhood phase, long time-lag between birth and age at first reproduction, relatively long brain development)^23^. Humans take twice as long as chimpanzees to reach maturity, and the reinitiation of active reproductive hormone production in the hypothalamus after years of inactivity produces accelerated growth at puberty that is not observed in our closest relative. Thus, the two main and unique traits of the human life cycle are the long period of growth (closely linked to the long human life-span) and the presence of a growth spurt in adolescence^23^. It is interesting to note that previous suggestions to explain the short stature of some human populations have been founded on opposite defining traits of the human life cycle: precocity versus a long growth period and absence versus presence of a growth spurt. Accepting the mechanisms suggested by previous studies would imply considering that the life cycle in Pygmies has departed from the characteristic life cycle of modern humans.

Whereas the focus of genetic studies so far has largely been on the ‘why' of the pygmy phenotype, how and when this phenotype is acquired during growth has seldom been investigated. The main obstacle in establishing patterns of growth is the lack of chronology^24^,^25^. To resolve this problem, previous studies on pygmies have been limited to a few individuals whose birth was recorded by researchers who monitored growth during the first 5 years^11^,^12^. Bailey reported height gain in the Efe and Jans followed up changes in weight in the Sua, all pygmy groups from East Africa, during the first 5 years of life. Both authors suggested that pygmies are smaller in size at birth compared with neighbouring Bantu groups. However, the period under study is not long enough to characterize growth in African pygmies.

Due to the lack of high-quality data, growth patterns in traditional societies are usually described in terms of the succession of mean values from a small sample with an assumed individual chronology^26^,^27^,^28^,^29^ or limited to a short period^30^. Although the overall shape of the growth pattern is similar among individuals in a given population, the heterogeneity in the levels and tempo of growth requires the use of a mixed modelling approach to describe, summarize and quantify the features and patterns of average growth in the population, using repeated measurements of each subject.

Here we present a high-resolution study on growth in Baka pygmies aiming to establish the pattern and tempo of acquisition of their short adult stature. The quantity and quality of the longitudinal data enable us to propose life history variables and a longitudinal growth model equivalent to those that exist only for industrial societies at present. Notably, growth in the Baka is characterized by the presence of growth spurts during adolescence, and they attain maturity at a similar age than other populations: the Baka therefore have the same human life cycle as all other human populations. We find that the short adult stature in the Baka results from a low rate of growth during the first 2 years of life, which produces a lasting delay in growth compared with European reference figures. Baka children are no different in size compared to other non-pygmy African populations during the first two years of life, but they become smaller from the age of 3 years. The Baka differ from the eastern pygmies (Sua and Efe) at birth, since newborns in the latter groups are already small in size. Differences in the growth process responsible for the pygmy phenotype in both eastern and western groups show a converging evolutionary pattern. A growth process that occurs early in life appears thus to play an active role in the acquisition of the pygmy phenotype and is responsible for adult morphological adaptation to environmental conditions.

## Results

### Baka populations

We collected life history data from Baka individuals to determine the pattern and tempo of acquisition of their short adult stature. During the 8-year period of our study in Moange-le-Bosquet, the Baka diet did not vary: their consisted of plants and animals from the forest even in the Catholic mission, where they had to employ a Baka man to cook every day. We observed a very marked change with the arrival of cheap alcohol in 2010. From this date, drunkenness became more common in the older men and now extends to adult women.

Although birth records exist, they do not give a clear idea of fertility or mortality rates, and even less of infant mortality. The contacts between nuns and pregnant women made it possible to record some births over the years, but the total number of births in groups around Le Bosquet remains unknown. Furthermore, since the Baka consult traditional healers more frequently than the health service, it is not possible to know whether the infant mortality records in the mission are representative of the population as a whole. An important factor affecting these variables is the mobility of groups. Although several families are often present at Bosquet, many circumstances (for example, hunting and gathering activities over a year, marriages, regular visits to other members of the same clan, availability of work, relationships with non-pygmies, relationships with nuns) have a direct influence on the presence/absence of people at Le Bosquet.

The growth pattern was obtained from the analysis of Baka individuals whose birth date had been recorded and whose parents were both Baka. The number of mixed couples of parents (Baka/non-Baka) is extremely low, with only 9 couples out of 346 in our records being mixed (2.6%). In eight of these cases, the man was a farmer and only in one case was the man a Baka married to a non-pygmy woman. Individuals were grouped into two cohorts: the first one covers from 0 to 3 years (girls=235, boys=246) and the second from 2 to 25 years of age (girls=271, boys=283; see Methods). The knowledge of birth dates allowed us to achieve a larger understanding of growth pattern with some life history variables involved in maturation. Growth pattern in Baka was compared with other studies conducted among Pygmies and non-Pygmies to check if processes of growth observed in Baka are also present in other Pygmy groups.

### Models of growth

The comparison of several models fitting weight and height to age shows that the Akaïke Information Criterion (AIC) for closeness of fit performed better with the Jenss–Bailey model of weight gain than the Reed model (5,355 versus 5,416) between birth and 3 years of age (period A). In period B (from 2 years to adulthood), the AIC performed better with the Count–Gompertz model than the Preece-Baines models for weight gain (7,013 versus 7,618) and height gain (7,159 versus 7,947; Supplementary Fig. 1)^31^. No trend over age appeared in the residuals in the resulting models; the residual standard deviations were around 1 kg and 1 cm, respectively (Supplementary Fig. 2). The models fit the data well and produce valid estimate of the average population trajectory (Table 1).

**Figure 1:**
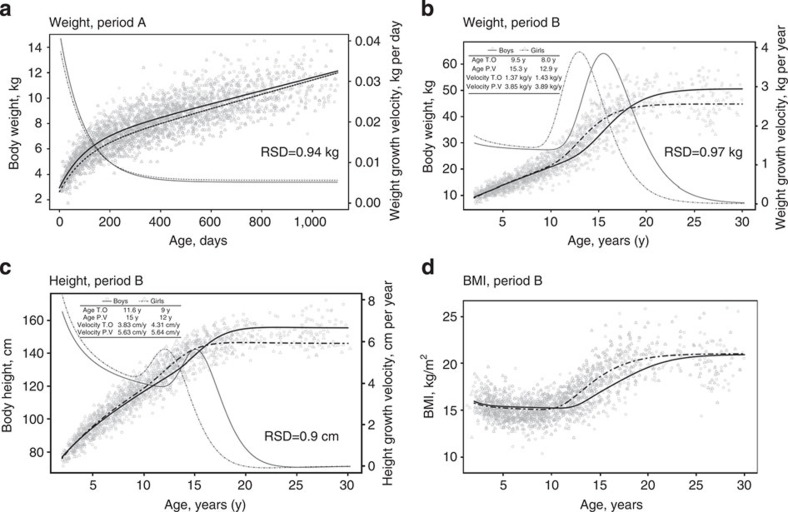
Growth trajectories in Baka pygmies. (**a**) Growth in weight during period A. (**b**) Growth in weight during period B. (**c**) Growth in height during period B. (**d**) Changes in BMI during growth. Growth trajectories show low residual standard deviations (RSD) and are different for boys (solid lines) and girls (dashed lines). Growth spurt in weight and height are clearly observable during adolescence and steady dimension are reached from 18 to 19 years in girls and 20 to 22 years in boys. T.O.: pubertal weight/height gain spurt, P.V.: peak weight/height growth velocity; BMI, body mass index.

**Figure 2:**
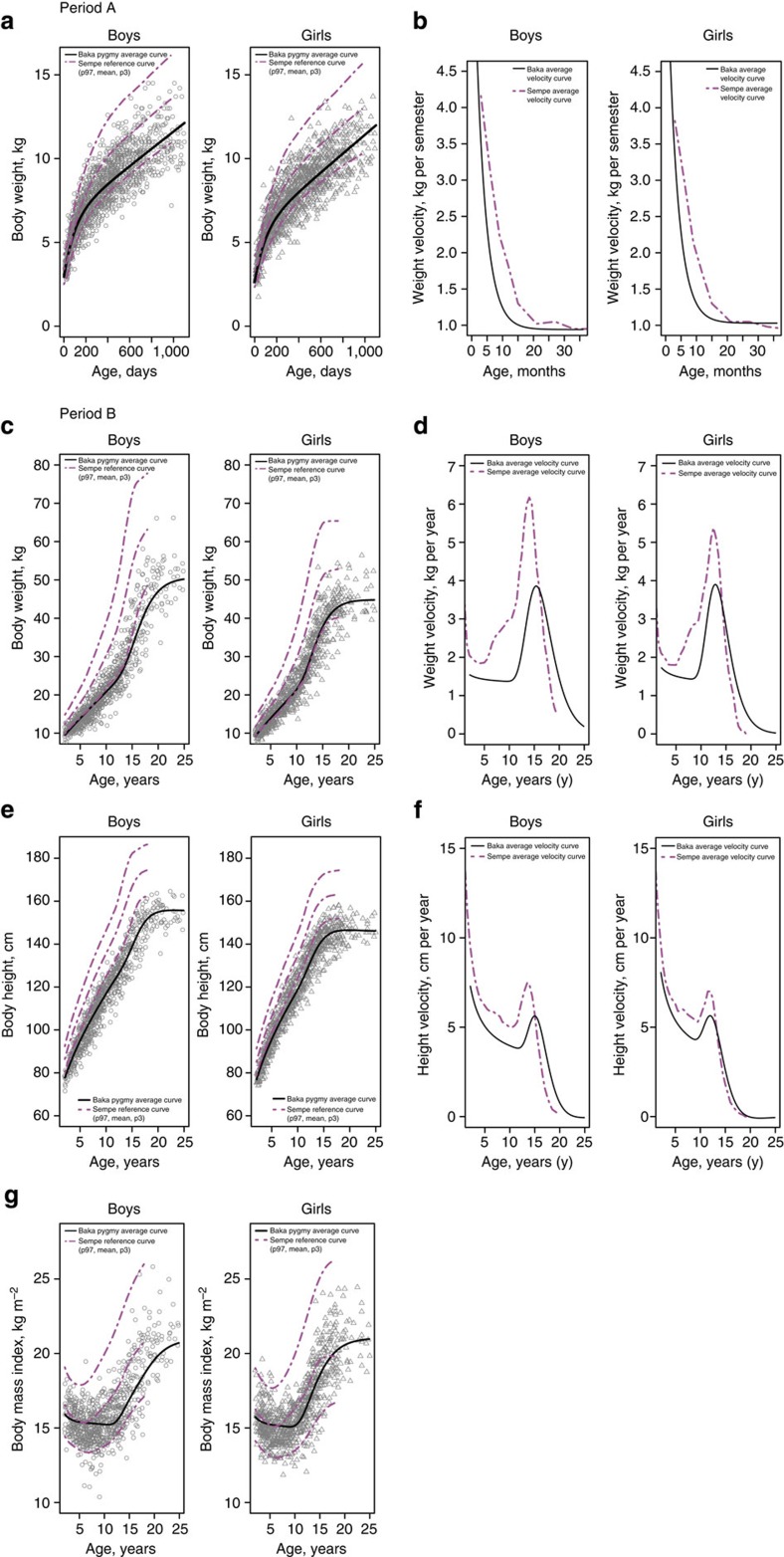
Growth curves for the Baka compared with standard French curves^32^. (**a**) Growth in weight during period A. (**b**) Weight velocity during period A. (**c**) Growth in weight during period B. (**d**) Weight velocity during period B. (**e**) Growth in height during period B. (**f**) Height velocity during period B. (**g**) Changes in BMI during growth. Baka curves show a markedly decrease from around the 3rd month to the age of 2 years and follow later the trajectory of lower percentiles even in the spurt. Adult BMI in the Baka reach about 21, which matches standard French values. BMI, body mass index.

The average growth curves for the Baka compared with European references are presented in Figs 1 and 2. Boys (*N*=246) are heavier than girls (*N*=235) during period A (from birth to 3 years of age) (Fig. 1a). Before 6 months, weight growth is faster in boys than in girls; afterwards, conversely, weight growth is faster in girls, whose weight almost catches up with that of boys by the end of period A. During period B (from 2 years of age to adulthood; Fig. 1b) the weight growth peaks at 12.9 years for girls (*N*=271) and 15.3 years for boys (*N*=283), with a corresponding rate of weight growth of 3.89 and 3.85 kg per year, respectively. The pubertal weight gain spurt begins at 8.0 years of age in girls (estimated weight=18.5 kg, s.d.=2.4) and 10.6 years in boys (estimated weight=22.1 kg, s.d.=3.4). Boys become heavier than girls from the age of 18 years. Weight become stable from the age of 19 years in girls (estimate weight=45.9 kg, s.d.=4.8) and 22 years in boys (estimate weight=52.9 kg, s.d.=6.6). Thus, from the start of the pubertal weight gain spurt to adulthood, girls gain around 27 kg in weight and boys around 30 kg. In girls (*N*=274), the peak of height velocity is 5.64 cm per year on average at the age of 12 years, and 5.63 cm per year on average in boys (*N*=275), 3 years later than in girls (Fig. 1c). The presence of peak velocity at puberty is not bound to the selected model. The start of the pubertal spurt in height gain occurs at 9.0 years in girls (estimated height=116 cm, s.d.=5.4) and at 11.6 years in boys (estimated height=122.8 cm, s.d.=5.7); this explains the higher values in girls from 10 to 16 years of age. Note that girls are taller than boys from the age of 5 years, finding that is uncommon but not unknown elsewhere^27^. Boys grow taller than girls from the age of 16 years. Adult height in girls is attained at around 18 years of age (estimated height=146.7 cm, s.d.=4.7) and at 20 in boys (estimated height=153.5 cm, s.d.=6.2). Girls and boys become about 30 cm taller from the start of the pubertal spurt in height up to adulthood. Therefore, our results clearly document the presence of a growth spurt in Baka pygmies. A body mass index (BMI) growth model was obtained from the model coefficients for predicted weight and height gain in period B (Fig. 1d). The BMI trajectory shows a similar pattern before the onset of puberty; after this, BMI in girls starts to increase earlier than in boys, who reach adult BMI values much earlier.

When our results are compared with standard French curves^32^, Baka curves for both sexes follow the French median weight during the first months after birth, but from around the third month to the age of 2 years their weight decreases markedly, coming close to the third percentile (Fig. 2a). The decrease in weight is due to a drastic reduction in the velocity of weight gain between those ages (Fig. 2b). From an age scale perspective, since velocity decreases much more rapidly than in standard populations it could be considered that development would be faster during the first months of post-natal life. In Fig. 2c we observe that weight in the Baka follows the third percentile until the end of adolescence, when it decreases in boys but increases in girls. The spurt in weight gain follows the standard pattern, although it is more markedly delayed in boys (Fig. 2d). The Baka height gain curve follows values lower than the third percentile in the standard curve (Fig. 2e). Growth in height increases by 1.83 cm per year in boys and 1.34 cm per year in girls, values that are below the third percentile in the standard growth curve (boys approximately 3.1 cm per year, girls approximately 2.1 cm per year; Fig. 2f). As for weight gain, the spurt in height shifts towards older ages in boys. Adult BMI in the Baka is reached at about 21 years of age (Fig. 2g), which matches standard French values. Unlike males, in whom BMI and skin fold thickness suggest a low body fat percentage until adulthood, the body fat percentage in girls increases during adolescence.

**Figure 3:**
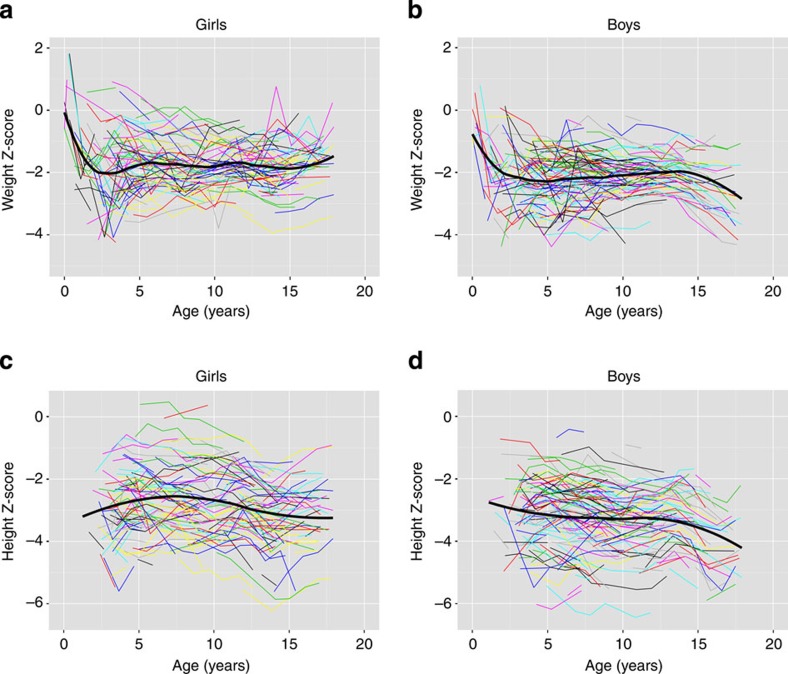
Spaghetti plots of individual Sempe^32^-based *Z*-scores of height and weight over time. Each colour line represents one subject. The plain black line represents the average trend in the population, thanks to a LOESS-based approach. The flexibility of the LOESS regression function was control through the smoothing parameter ‘span' set at 0.3 for weight and 0.8 for height. (**a**,**b**) *Z*-score of weight at birth in boys and girls from the Baka population is close to zero, which means that weight at birth is not very different from the Sempe reference one. Then, the mean *Z*-score decreases rapidly until 2 or 3 years and remained around −2 until the end of follow-up in girls and until 15 in boys where mean *Z*-score seems to decrease again. (**c**,**d**) Mean *Z*-score of height (available from 2 years), is already at about −3 at this age and fluctuates around this value along the follow-up. In boys, as for weight, height difference seems to increase a little from 15 years.

The plot of individual Sempe^32^-based *Z*-scores of height and weight over time (Fig. 3) shows that Baka birth with a weight close to standard values and decrease markedly during the first month of life becoming around −2 *Z*-scores from 2 to 3 years until adulthood in girls; in boys, difference seems to increases from 15 years of age. Data on height in Baka for earliest years of post-natal life are scarce and does not allow to obtain the mean *Z*-score but height is already at about −3 at 2 years and fluctuates around this value along the follow-up; as to weight, difference in height seems to increase from 15 years in boys. Results in Fig. 3 clearly show that primarily difference in dimensions is established early in life, certainly during infancy (Fig. 3a,b).

### Life history variables

**Figure 4:**
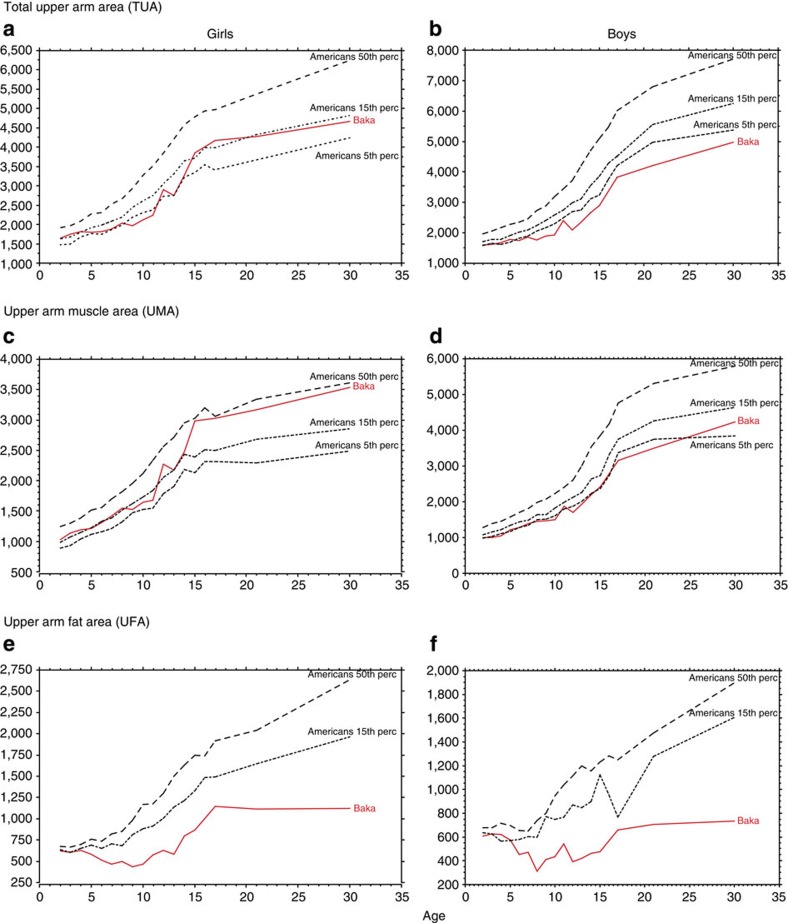
Upper arm indicators of body composition in Baka children. The mid upper arm circumference combined with triceps skinfold measurements allows parameters for muscle (protein) and fat (energy) stocks to be computed^58^. (**a**,**b**) Compared to American references, the values for Baka girls are always higher than for Baka boys; the relatively bigger arm in girls is mainly due to greater muscle development. (**c**,**d**) In girls, UMA is close to the American average after puberty, while in boys it remains below the 10th American percentile. (**e**,**f**) Conversely, fat is very low in both sexes, with values close to those of Americans in the early years, but a marked decrease during childhood (from 3 to 6 years of age); there is no fat accumulation as the children become older. In other words, Baka children are muscular and lean.

Baka children are very lean from an early age, but their muscular mass develops considerably, especially, in girls at puberty who display values close to the American reference^33^ (Fig. 4).

The median age at menarche was 14.5 years (95% CI=14–15; *N*_individuals_=128; *N*_observations_=199), which is within the range of variation of modern populations^34^ (Supplementary Table 1). Evidence of menstruation was recorded as early as 11 years of age, and all girls were menstruating by 17 years of age. In well-nourished British girls the menarche usually occurs 6 and 12 months^23^ after the peak of the growth spurt. This was not observed in the Baka, in whom the menarche occurs 2.5 years after the growth spurt, probably because of the low level of body fat. The earliest age at first pregnancy in the Baka was 16 years (modal; Table 2). First reproduction in males seemed to occur later, at 20 years of age (modal value; Table 2). Neither of these values are especially early, as has been claimed, when compared with other forager and non-forager populations^35^,^36^. The median age at first birth for all Baka women is about 18 years (*N*=28), which is about 1 year less than the mean for other groups of forager and non-forager women living in premodern societies^23^,^36^.

The median interbirth interval was 34.5 months (*N*=443), with the modal in the 30–35.9 months class (2.50–2.99 years; *N*=121; Table 2). In half of cases (220 out of 443), the interval was between 2 and 3 years, reflecting abstention from sexual intercourse for >1 year. In seven out of eight cases where the interbirth interval was <18 months, the first offspring died during the first year of life. These values for the Baka are similar to those reported for many developing countries^37^.

Our results suggest that the pygmy phenotype in the Baka cannot be associated with early cessation of growth^22^ or to the lack of an adolescent growth spurt^20^, since the Baka growth pattern conforms to the standard human life cycle. Our results show that the rate of growth decreases markedly from birth to 2 years of age, after which it follows a growth pattern close to that observed in lower percentiles in other groups. The low adolescent growth spurt corresponds to low values in the standard growth curve, and the final adult stature maintains the gap already established during infancy.

## Discussion

Comparisons of our results with other African (pygmy and non-pygmy) populations reveal a number of interesting points. The Baka are significantly heavier than Sua pygmies from East Africa in almost all age classes, and their weight is similar to that of the Bantu from the Ngayu region with the highest socioeconomic status (Tables 3 and 4, Supplementary Fig. 3). The Baka are taller than Efe pygmies in all age classes and similar in height to the Lese in the younger age classes. When comparing Baka sizes with non-pygmy African populations (Table 5, Supplementary Table 2, 3 and 4), it is worth noting that the Baka are no smaller at the perinatal stage, and that in several cases, the Baka are heavier than other populations, such as the Datoga and Keneba. Smaller sizes in the Baka are observed from the age of 2–3 years.

Except in Bailey and Jans^11^,^12^, all other studies on growth in African pygmies were carried out with a few individuals with cross-sectional data and without real chronology^26^,^27^,^28^,^29^. The difference in the quality (that is, longitudinal study of individuals of known age) and in the quantity (that is, several hundred individuals from 0 to 25 years of age) of data used in the present study in relation to previous ones (that is, cross-sectional data for a smaller number of individuals of unknown age) makes comparison difficult: the differences between our results and those from other groups could be due to errors in age estimations or to methodological bias (cross-sectional study), even if differences between groups cannot be ruled out.

In a study on growth and life-history traits in small-scale societies, Walker *et al.*^25^ suggested that African pygmies are characterized by relatively fast child-juvenile growth for their adult body size, with relatively early puberty, menarche and first reproduction. They founded their analysis on bibliographical data. Specifically for Baka pygmies from Cameroon, they used data provided by Yamauchi *et al.*^38^. The values for adult size^38^ are close to those of this study, as are age at menarche and average age at first reproduction^25^, which are not earlier than in other populations. If an average child-juvenile growth velocity is obtained following the same method used by Walker *et al.*^25^, that is, difference in size from age 3 years to age 10 years divided by 7 years, the values for the Baka are lower than those obtained by Walker *et al.* and do not support their conclusion of fast child-juvenile growth relative to body size in the Baka. This neither fast nor slow childhood-juvenile growth concurs better with other life history variables (for example, age at menarche and age at first pregnancy), which occur at similar ages among the Baka as in other populations. Differences in the results from our work and that of Walker *et al.* can be explained by the observation of Walker *et al.* that age estimation for African pygmies is ‘most uncertain' (page 299), and thus introduces a large margin of error (>14%). Furthermore, Walker *et al.* used published data which they could not check for reliability: for instance, they use data from Yamauchi *et al.*^38^, but Yamauchi *et al.* measured adults and only six individuals under 20 years of age (page 71); Walker *et al* certainly used another source for the Baka but this is not provided.

Walker *et al.*^25^ related the fast child-juvenile growth in pygmies to juvenile mortality, but, no source is given to support high mortality in the juvenile period; Yamauchi *et al.* do not give any information about mortality. Mortality rates are very difficult to obtain, first of all because the real chronology of individuals is unknown: even at Bosquet, the estimated age of individuals older than 25 is uncertain and the uncertainty increases with age. Mobility also adds to the difficulty of building up reliable records. If we consider the few records and oral communications available from Bosquet, high mortality mainly seems to concern infancy and early childhood.

Mean age at first reproduction and interbirths interval have been reported for other forager groups^39^. The median age of 19–20 years at first reproduction reported for the Ache and !Kung suggests that it occurs later than in the Baka. The interbirth intervals for the Ache and !Kung (forager mean=41.3 months^39^) are higher than for the Baka. Differences between the Baka and other forager groups may result from the use of indirect age estimation methods (absolute chronology was unknown to Ache and !Kung), although some variation between forager societies, probably resulting from adaptive strategies, cannot be ruled out.

Different growth processes during development have been proposed to explain small adult phenotypes. The presence of small bodied populations in different continents suggests that they acquired their small body phenotype independently; even in Southeast Asia, small adult stature was probably acquired more than once^22^. If the small body phenotype was indeed acquired independently in different regions, this does not imply that it was acquired by similar mechanisms indicating parallel evolution. Parallel or convergent evolution can be assessed by comparing the growth process in two populations with a small body size. If a precocious arrest of growth is the cause of small body size in the Aeta^22^^,contra^^40^ while the pygmy phenotype is produced by a prenatal mechanism in the Efe, as suggested by the size at birth^22^, this would indicate convergent evolution in the Aeta and Efe towards a small adult size. On African pygmies, Perry *et al.*^41^ have recently suggested convergent evolution between the eastern and western groups towards the pygmy phenotype, although it is not clear if different degrees of gene expression can be interpreted as convergent evolution. Concerning growth processes, Merimee *et al.*^42^ suggested that the pygmy phenotype in the Aka is due to the absence of a growth spurt. Bailey's and Merimee's results cannot be compared to assess whether the pygmy phenotype in the Aka and Efe is acquired through different mechanisms, since Bailey studied growth in size from 0 to 5 years of age whereas Merimee analysed the IGF1 concentration in adolescence; the two processes are not mutually exclusive and could be present together in both groups. Our results show that the Baka do not differ in size at birth from non-pygmy African populations and are larger at birth than the Efe and Sua. In other words, while the small adult stature in eastern pygmies (Efe and Sua) is the result of a particular growth pattern during prenatal life, the pygmy phenotype in the Baka results from a slower rate of growth during infancy. The pygmy phenotype is thus acquired through different growth mechanisms in East and West Africa, which points to convergent evolution.

Knowledge of growth processes in pygmy populations is crucial to understanding their evolutionary history. The presence of similar growth patterns in eastern and western pygmies does not inform about the process of acquiring the pygmy phenotype, since this could have occurred before or after the split into two clusters. The presence of convergent evolution, however, indicates that development of the pygmy phenotype occurred after the split into two clusters (eastern and western) at around 20,000 BP^13^.

Assuming that the pygmy phenotype is an adaptation to life in the rain forest, the process governing its acquisition has to be closely linked to the history of the environment. On the basis of our results, on palaeoclimatic data and on genetic studies^13^,^14^,^43^, one can hypothesize^44^ that at around 20,000 years BP, a population living around the periphery of the rain forest moved eastwards and westwards to follow the forest's regression and the expansion of the savannah, which at this point was nearing the Equator as a consequence of climatic changes associated with the OIS2 global cooling event^45^. With the advent of warmer conditions at the end of the Pleistocene (after 13,000 BP) and the extension of the rain forest until 3,000 BP^46^,^47^,^48^, populations inhabiting areas closer to the forest during OIS2 probably could not move away due to demographic pressure or, in more recent times, the expansion of agricultural activity, and had to adapt to life in the expanding rain forest. From this point of view, hunter-gatherer behaviour related to the rain forest began at that moment. Gene flows between the eastern and western populations stopped and biological adaptation to new environmental conditions developed independently in the eastern and western equatorial rain forest.

Humans wean relatively early compared with chimpanzees, bonobos and orang-utans and Bogin^49^ proposed that human weaning by 30–36 months is a life history trade-off for more rapid birthing by women. In humans, weaning is associated with the transition from infancy to childhood. Hochberg and Albertsson-Wikland^50^ have suggested that the infancy-to-childhood transition represents an adaptive evolutionary strategy of growth plasticity to match environmental cues and energy supply. The adaptive evolutionary strategy in the Baka may have been downshifted to occur in infancy. If the pygmy phenotype is indeed an adaptation to environmental conditions, then the foundations of this adaptation may be laid at a very early stage in postnatal growth. Our results suggest that genetic and endocrine processes acting during infancy are involved in establishing differences between pygmy and non-pygmy groups by adapting adult morphology to environmental pressure. Great plasticity of growth is certainly closely linked to the duration of growth^49^ and growth in *H. sapiens* is longer than any other hominid species^51^. *Homo sapiens* could therefore be characterized by its high capacity for growth plasticity during infancy. This capacity, which may be unique to our species, may have played a fundamental role in the biological adaptation that enabled its worldwide expansion and occupation of dissimilar environments within a short period after moving out of Africa.

## Methods

### Sample size and data

In the Moange-le-Bosquet locality in Southeast Cameroon, nuns with medical training recorded births of Baka pygmies from the 1970s. They weighed new-born infants and measured their weight from birth to 3 years of age. The birth records from the 1970s and early 1980s have been lost, but those from 1988 were available to us. In addition, from 2007 and each year for 8 years, with the informed consent of the head of the village and the informed consent of all individuals, we systematically measured the height and weight of Baka individuals of known age living in the locality. This study obtained approval of the Centre National de la Recherche Scientifique, Agence National de la Recherche (France) and Institut de Recherche et Développement and was carried out as part of the international agreement between the IRD and the Ministry of Scientific Research and Technology of Cameroon. The Baka at Bosquet are semi-nomadic, spending long periods in the forest to gather produce or look after small plantations. For this longitudinal growth study, a round trip to visit the nearest camps (up to 12-km away) was made every year to measure individuals. All the Baka who came to us were measured, but only those whose birth date had been recorded by the nuns and whose parents were both Baka were included in the study. The age of each individual is given in days.

Growth in the Baka is described from the analysis of growth during two periods corresponding to two cohorts of the Baka population: the first period (A) covers birth to 3 years and the second (B) covers 2 to 25 years of age. For period A, the weights of 235 girls and 246 boys were measured by the nuns every 3 months from 1985 to 2007, with a total number of measurements per subject ranging from 1 to 26 and a median of 5 (Table 6). Weights were measured to the nearest 0.1 kg. Period B analyses were made for 557 individuals: weight gain was monitored in 283 boys and 271 girls and height gain in 275 boys and 274 girls from 2007 to 2014. Thus, the maximum number of measurements per subject was 8, homogenously covering the age range, and at least two measurements were recorded for 50% of individuals (Table 6). Weights were measured with electronic scales (Tanita) to the nearest 0.1 kg and height with a steel height gauge to the nearest 0.1 cm. With this protocol, each subject contributed to the average population growth only for a short period of time, but the inclusion of subjects of different ages enabled us to cover the period from two years of age to adulthood.

### Models of growth

The data were used to build separate growth models for each period. For a given period, we fitted one model for weight and one model for height (period B only): boys and girls were included in the same model, with fixed gender-specific coefficients to have gender-specific mean growth trajectories. We used structural non-linear models to fit the growth data because the model parameters can be meaningfully interpreted and because milestones and BMI can be easily calculated^52^.

When using growth models, it is assumed that all the children in a population share a similar pattern of growth in weight and height, respectively, which is reasonable in practice. Furthermore, these models allow some variations at the individual level in terms of the degree and timing of growth. Linear models usually cannot accurately describe growth over a long period; structural mathematical equations have therefore been proposed to describe the shape of the average growth curve. The complexity of the models varies according to the period and the age range considered. The Jenss and Reed models both give a good description of growth in weight and height from birth to 5 years, whereas Count–Gompertz, Preece-Baines and Bock–Thissen-du Toit are those most often used to model growth from early childhood (2 years for Preece-Baines) up to adulthood.

The Jenss–Bayley growth curve model (equation 1) fits growth data from birth to six or eight years of age, before growth starts to accelerate again (at the start of puberty). It was recently described in detail in a paper on growth modelling^31^. The equation to model the *j*^th^ growth measurement of the *i*^th^ subject, with *y*_*ij*_ being weight or height and *t*_*ij*_ the age, can be expressed as in equation 1 below or reparameterized according to equation 2 to obtain birth characteristics and improve convergence.


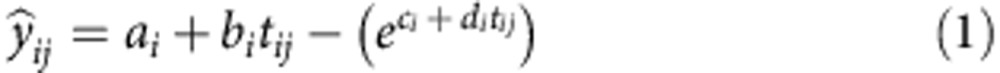



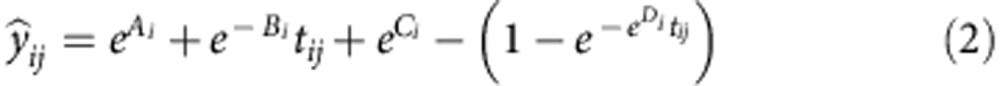


The mean growth trajectory is obtained from the fixed component estimate of the model 2 coefficients. Briefly, *e*^*A*^ is an estimate of birth weight, *e*^*−B*^ is an estimate of the weight gain rate in the oldest individuals (3 years in this case), *e*^*C*^ can be interpreted as the degree of the growth spurt and *e*^*D*^ allows modelling of the growth curve for the earliest ages (shape of growth deceleration). The Jenss-Bayley model is differentiated to give the growth rate (Table 1).

The Count-Gompertz growth model is a six-parameter function used to describe growth from birth to adulthood:


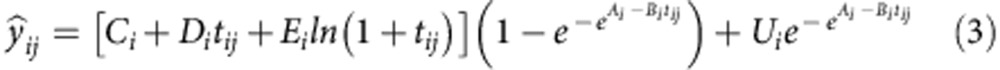


Where *y*_*ij*_ is the weight or height at the *j*^*th*^ growth measure of the *i*^*th*^ subject, and *t*_*ij*_ is the age. The parameter *U* can be set as the final size observed in each individual curve. This model has a childhood component described by the Count function (parameters C, D and E) and an adolescent component described by the Gompertz function (parameters A and B and C) (Table 1).

More recently, models such as the cubic spline^53^ or fractional polynomial models^54^ have also been proposed. As opposed to some other published growth equations, fitting cubic spline and fractional polynomial models produces parameters that cannot easily be interpreted but, as they are more flexible, they usually fit the data better, although they can carry a risk of over-fitting in some circumstances. They can therefore lack robustness, especially if the numbers of knots or polynomials increases, when they can behave oddly at extremes and may be significantly influenced by outliers. A more robust method, SITAR^55^, based on the shape invariant model^56^, has also been recently developed for growth modelling.

All these growth models can be hierarchically fitted using mixed-effect models to take repeated measurements into account to estimate the average growth curve. This allows missing data to be taken into account and does not require the same age at measurement for all individuals.

For period A, the Jenss-Bayley and Reed models, both known to fit growth data accurately up to 6 years of age, were compared. To fit the data from two years to adulthood (period B), the Count-Gompertz and the Preece-Baines models were compared. For both periods, the model fits were compared using the AIC defined as −2 (log-likelihood) +2 (number of calculated parameters), with a lower AIC indicating a better fit. The modelling was performed using the ‘nlme' R software package^52^. The R function fits the nonlinear mixed-effects model with the formulation described in Lindstrom and Bates^57^, which uses a Newton-Raphson algorithm to estimate the parameters. To fit a non-linear model, initial values are required. First, we fitted a fixed-effect model with the R ‘nls' function. To choose the initial parameters, we used published data and other initial values by trial and error. We used different starting values and introduced random effects one at a time to limit the risk of obtaining local maxima for the likelihood function. We checked the validity of the model by analysing the trend in the residuals against time. We also checked that the weight or height velocity peak at puberty was not bound by the selected model, by again fitting the data using the SITAR method based on a random-effect cubic spline model and developed by Cole *et al.*^55^. This method fits the average growth curve by a spline model, which does not impose a particular shape and allows the introduction of random effects to take longitudinal data into account. For girls, the average growth and velocity trajectories were very similar (Supplementary Fig. 4). The SITAR model failed to fit the data for boys (probably because there were relatively less data on young adults), but we can expect similar average curve modelling. Once the average model fits the data, many population characteristics of the curve can be obtained, including predictions of weight and height as well as growth velocities at given ages and age at the peak of height growth velocity.

The BMI growth trajectory life follows a non-monotonic pattern (BMI generally increases in the first year, then decreases before increasing again during childhood) and is thus more complex to model than weight and height growth trajectories. Using individual weight and height equations, an equation for BMI over time can be obtained. BMI was calculated as the ratio of the geometric mean weight over the squared geometric mean height. For boys, the number of BMI measures was lower during adulthood than during childhood and adolescence so that the imprecision produced on the BMI growth curve for these ages is probably higher.

Because the growth models obtained for the Baka do not exist for any other sub-Saharan populations, they were compared with Sempe's curve for a French population based on a longitudinal study^32^.

### Life history variables

Body composition was assessed by limb circumferences and skinfold thickness. The midupper arm circumference (MUAC) combined with triceps skinfold thickness enabled us to estimate muscle mass and subcutaneous fat^58^ in 379 individuals from childhood to adulthood; adult over 20 years of age but with no known birth date were also included and grouped into 10-year age classes. Three main indicators are considered: total upper arm area (TUA), muscle area (UMA) and fat area (UFA). The calculations are made according to Frisancho's formulae^58^

- TUA=MUAC^2^/4*π*
- UMA=(MUAC/*π*−triceps)^2^ × *π*/4
- UFA=TUA−UMA

The reference data for MUAC, triceps skinfold measurement and UMA are from white Americans who participated in the Ten-State Nutrition Survey in 1968–1970 (ref. 58).

The birth records allowed us to obtain several life history variables, which, in conjunction with other traits from the model developed for the Baka (for example, age and amplitude of the growth spurt) are of great importance to current discussions on the evolution of growth patterns in modern humans. Individuals whose birth was recorded many years ago have recently appeared in the records as parents, enabling us to obtain the age of first reproduction for 28 girls and in 10 boys. The interbirth interval was calculated in 443 cases (886 births); twins were recorded in only three cases. During our time in Le Bosquet, a Baka girl carried out interviews to determine the presence or absence of menstruation, obtaining 199 answers from 128 girls of 10 to 17 years of age, which were analysed with probit analysis to estimate the median age at menarche.

### Comparison with African groups

Since it is more appropriate to compare the Baka with other traditional societies, growth in the Baka was also compared with patterns in other African populations. With the exception of Gambian individuals, with whom a long project on growth started in the late 1940s (ref. 59), growth in sub-Saharan African populations is known from cross-sectional studies on individuals of unknown age. The irregular, infrequent or time-limited studies, and especially the lack of known individual chronologies, have led authors to hypothesize about growth from sporadic data and forced them to develop analyses to deduce life history variables such as age at first pregnancy or age at menarche, among others. It should be noted that contrary to previous studies, our results are based on the analysis of individuals of known age who were monitored for 8 years. The life history variables are therefore known from raw data and no calculations were necessary to make assumptions on these variables.

Several studies have focused on growth in pygmies, but the majority are founded on cross-sectional data from a few individuals of unknown age^25^. In contrast, studies by Bailey^11^ and Jans^12^ are the only ones addressing growth in pygmies on the basis of longitudinal data with an exact, although short chronology (5 years). In both the cases, the authors were present at births and followed the growth of infants from birth to 5 years of age. Bailey measured height in the Efe and Lese, the neighbouring Bantu group in close contact with the pygmy group. Jans measured weights among the Sua and in different social–economic categories among neighbouring Bantu. The populations to which the Efe and Sua were compared in the original study were also used for comparisons with the Baka. Giving Baka ages in days enabled us to form age classes following the criteria used in previous studies for other groups, and thus facilitated comparisons. Because previous studies have only published average values, significance was assessed using one sample *t*-test (StatView).

## Additional information

**How to cite this article:** Ramirez Rozzi, F. V. *et al.* Growth pattern from birth to adulthood in African pygmies of known age. *Nat. Commun.* 6:7672 doi: 10.1038/ncomms8672 (2015).

## Supplementary Material

Supplementary Information Supplementary Figures 1-4, Supplementary Tables 1-4 and Supplementary References

## Figures and Tables

**Table 1:** Description of the parameters from the growth curve models.

	**Parameters**	**Boys**	**Girls**	***P*** **value**
		**Mean (95% CI)**	**Mean (95% CI)**	
Cohort A: weight (kg) growth modelling		*N*=246	*N*=235	
	A	1.08 (1.04–1.12)	0.97 (0.90–1.03)	0.001
	B	5.27 (5.21–5.32)	5.18 (5.12–5.21)	0.03
	C	1.26 (1.20–1.31)	1.15 (1.11–1.18)	0.005
	D	4.60 (4.48–4.71)	4.55 (4.37–4.72)	0.2
	RSD	0.94		
Cohort B: weight (kg) growth modelling		N=283	N=271	
	A	5.86 (5.29–6.43)	5.66(5.09–6.23)	0.0001
	B	0.36 (0.32–0.40)	0.41 (0.37–0.46)	
	C	6.01(5.19–6.83)	5.04 (4.18–5.90)	
	D	1.30 (1.13–1.46)	1.27 (1.04–1.50)	
	E	0.74 (0.11–1.39)	1.40 (0.32–2.48)	
	U	50.6 (48.8–52.4)	44.8 (43.7–46.0)	
	RSD	0.97		
Cohort B: height (cm) growth modeling		*N*=275	N=274	
	A	7.35 (6.39–8.32)	5.89 (5.34–6.43)	0.0001
	B	0.45 (0.38–0.51)	0.43 (0.39–0.48)	
	C	55.8 (53.0–58.6)	52.2 (49.2–55.2)	
	D	2.66 (2.32–2.99)	2.53 (2.08–2.96)	
	E	14.4 (12.0–16.8)	17.1 (14.3–20.0)	
	U	155.4 (154.1–156.7)	146.0 (144.9–147.0)	
	RSD	0.90		

CI, confidence interval; RSD, residual standard deviation.

Data from boys and girls fitted in one model allowing gender-specific parameters. *P*-value for the test of the difference between boys and girls (Cohort A: Student *t*-tests; cohort B: maximum likelihood ratio test for global difference).

Cohort A: Jenss growth curve model 
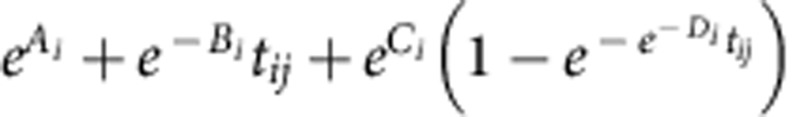
; *e*^A^ gives an estimate of birth weight, *e*^*−*B^ gives an estimate of weight growth velocity at the highest ages (3 years here), *e*^C^ can be interpreted as the degree of growth spurt and *e*^D^ allows to model the curvature of the growth during the earliest ages (growth deceleration).

Cohort B: count Gompertz growth curve model 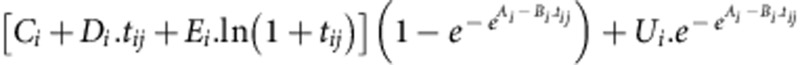; *U* is an estimate of the final average size. This model has a childhood component described by the Count function (parameters *C*, *D* and *E*) and an adolescent component described by the Gompertz function (parameters *A*, *B* and *C*).

**Table 2:** Life history variables in Baka.

**Interbirth interval**			**First pregnancy**	
**Midpoint (years)**	**Age class (years)**	***N***	**Midpoint (years)**	***N***
1.25	1–1.49	8	16.5	10
1.75	1.5–1.99	25	17.5	6
2.25	2–2.49	99	18.5	7
2.75	2.5–2.99	121	19.5	1
3.25	3–3.49	57	20.5	3
3.75	3.5–3.99	29	21.5	1
4.25	4–4.49	28	Total	28
4.75	4.5–4.99	20	**First paternity**	
5.25	5–5.49	15	Midpoint (years)	*N*
5.75	5.5–5.99	10	18.5	2
6.25	6–6.49	7	19.5	2
6.75	6.5–6.99	4	20.5	5
7	>=7	20	21.5	1
Total		443	Total	10

**Table 3:** Comparison of height growth between the Baka, the Efe and the non-pygmy neighbours the Lese.

**Girls and boys together**											
**Age in years**	Baka*			**Efe**^11^			**Lese**^11^			**Baka versus Efe**	**Baka versus Lese**
	***X***	***N***	**s.d.**	***X***	***N***	**s.d.**	***X***	***N***	**s.d.**		
1.5	72.5	7	3.4	66.5	18	2.3	71.6	18	4.8	*** B	0.808
2	77.0	19	2.9	72.1	19	2.5	76.1	18	2.7	*** B	0.521
2.5	79.0	28	3.2	75.5	23	3.4	82	21	3.1	*** B	***
3	83.4	46	3.1	78.6	13	3.2	84.9	20	4	*** B	***
3.5	86.8	60	3.8	82.8	12	2.4	88.3	22	4.1	*** B	***
4	89.3	69	4.4	86.2	10	3.6	91.4	14	4.7	*** B	***
4.5	92.8	60	4	87.4	9	3.6	93.2	13	4.7	*** B	0.470
5	94.9	66	5.2	89.9	11	2	96.0	11	6	*** B	0.080

B, Baka values are higher.

***=*P*<0.001, **=*P*<0.01, *=*P*<0.05 (one sample *t*-test).

^*^This study.

**Table 4:** Comparison of weight growth between the Baka, the Sua and the non-pygmy neighbour the Bantu from Ngayu region.

**Girls and boys together**									
**Age in months**	Baka*			**Sua**^12^		**Bantu**^12^†		**Baka versus Sua**	**Baka versus Bantu**
	***X***	***N***	**s.d.**	***X***	***N***	***X***	***N***		
Birth	3.00	2		2.64	40	2.97	NA		
1	4.04	25	0.58	3.64	43	4.03	NA	** B	0.9406
4	6.11	22	0.82	5.62	48	6.23	NA	** B	0.5124
7	7.01	18	1.12	6.52	45	7.32	NA	0.0815	0.2515
12	7.89	14	1.15	7.46	42	8.27	NA	0.1839	0.2431
18	9.21	84	1.16	8.42	25	9.20	NA	*** B	0.955
24	10.20	47	1.35	9.24	22	10.06	NA	*** B	0.4794
30	10.77	24	1.32	10.16	14	11.05	NA	* B	0.3031

***P<0.001, **P<0.01, *P<0.05 (one sample *t*-test).

^*^This study.

^†^Only the subsample with good diet was kept for comparison. B, Baka values are higher.

**Table 5:** Comparison of height growth between the Baka and non-pygmies African groups.

**Girls and boys together**											
**Age in months**	Baka*			**Bagandu**^24^			**Bamileke**^24^			**Baka versus Bagandu**	**Baka versus Bamileke**
	***X***	***N***	**s.d.**	***X***	***N***	**s.d.**	***X***	***N***	**s.d.**		
15	71.4	6	3.4	73.9	14	3.5	72.0	36	3.5	0.1322	0.6904
21	76.0	11	3.9	79.1	14	2.6	75.1	20	5.5	*	0.4722
27	77.6	25	2.5	83.7	4	2.4	79.5	28	4.7	***	***
33	81.7	29	3.6	87.4	16	2.9	85.1	19	3.6	***	***
39	84.6	66	3.5	90.4	14	4.3	87.9	18	4.8	***	***
45	88.5	56	4.2	95.3	9	3.6	90.6	17	3.9	***	***
51	90.4	66	4	97.6	7	4.7	92.9	13	3.6	***	***
57	94.3	60	4.3	102.1	15	3.1	97.1	18	5.4	***	***
63	95.8	65	4.8	105.0	2	1	99.8	12	6.2	***	***
69	99.8	64	4.8	104.5	13	4.9	102.0	4	2.5	***	***

***P<0.001, **P<0.01, *P<0.05 (one sample *t*-test).

^*^This study.

**Table 6:** The Baka data sets.

**Cohort A (from 0 to 3 years of age)**												
**Gender**	***N***	**Measure**	***N***	**Min**	**1stq**	**Med**	**3rdq**	**Max**	<**5 Measures (%)**	**At least one measure (%)**		
	**ind**		**mes**							**0–1 y**	**1–2 y**	**2–3 y**
Girls	235	Weight	1,491	1	3	5	9	26	42.10	93.2	66.4	29.0
Boys	246	Weight	1,598	1	3	5	10	20	42.10	95.5	64.4	28.0

**Cohort B (from 2 to 20 years of age)**												
**Gender**	***N***	**Measure**	***N***	**Min**	**1stq**	**Med**	**3rdq**	**Max**	**At least one measure (%)**			
	**ind**		**mes**						**2–5 y**	**5–10 y**	**10–15 y**	**15–25 y**
Girls	271	Weight	878	1	1	2.5	5	8	37.8	43.3	35.6	25.0
	274	Height	865	1	1	2	5	8	37.1	43.2	33.6	24.7
	271	BMI	865	1	1	2	5	8	37.1	43.2	33.6	24.7
Boys	283	Weight	879	1	1	3	5	8	35.4	48.9	33.2	22.5
	275	Height	866	1	1	3	5	8	34.5	49.8	33.5	22.9
	275	BMI	866	1	1	3	5	8	34.5	49.8	33.5	22.9

BMI, body mass index; Max, maximum; Med, median; Min, minimum; mes, measurements; ind, individuals, q, quartile, , y, years.
