# Supplementary material for: Growth pattern from birth to adulthood in African pygmies of known age
Source: Nat Commun. 2015 Jul 28;6:7672. doi: 10.1038/ncomms8672 (PMC4525207; doi:10.1038/ncomms8672)
Supplement: Supplementary Information — Supplementary Figures 1-4, Supplementary Tables 1-4 and Supplementary References [file ncomms8672-s1.pdf]

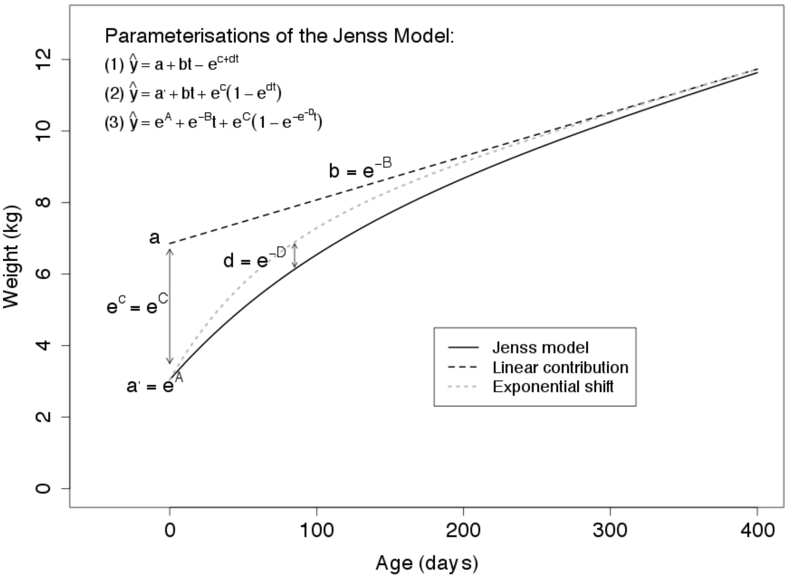

Supplementary Figure 1: Characteristics of the Jenss model in term of interpretation of the coefficients. (1) is the historical parameterisation. Parameterisation (2) allows e^{A} to be interpreted as the measurement at birth (length only when considering neonatal weight loss). Parameterisation (3) adds positivity constraints to the parameters to facilitate convergence of the model.

a.

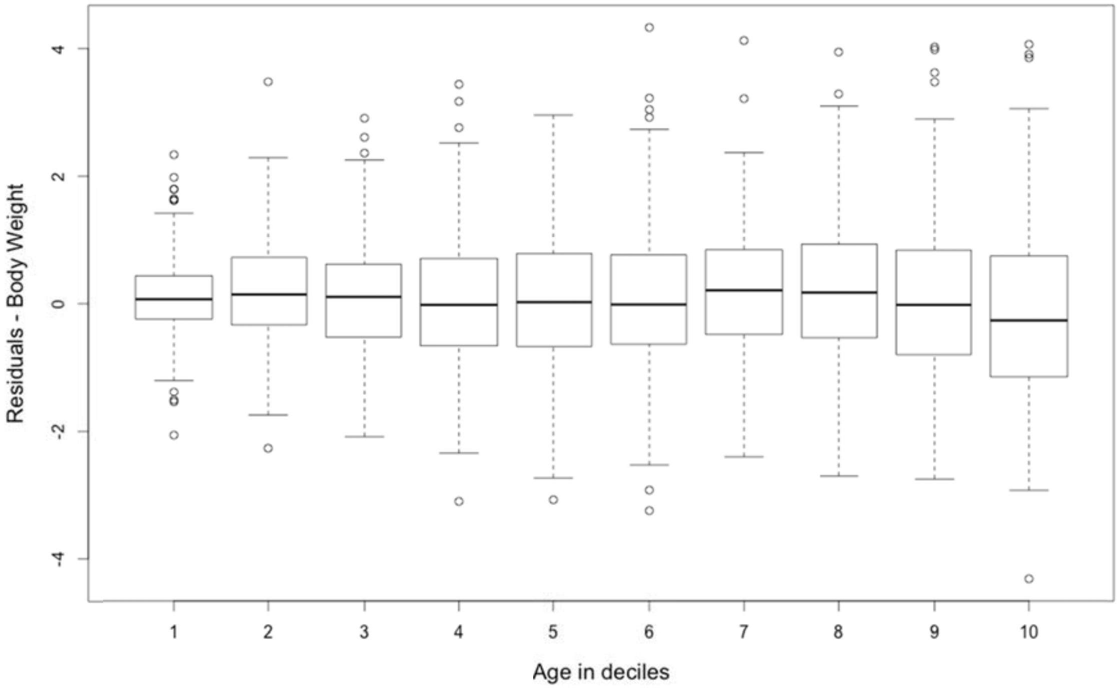

Residuals - Body Weight
4
2
0
-2
-4
1
2
3
4
5
6
7
8
9
10
Age in deciles

b.

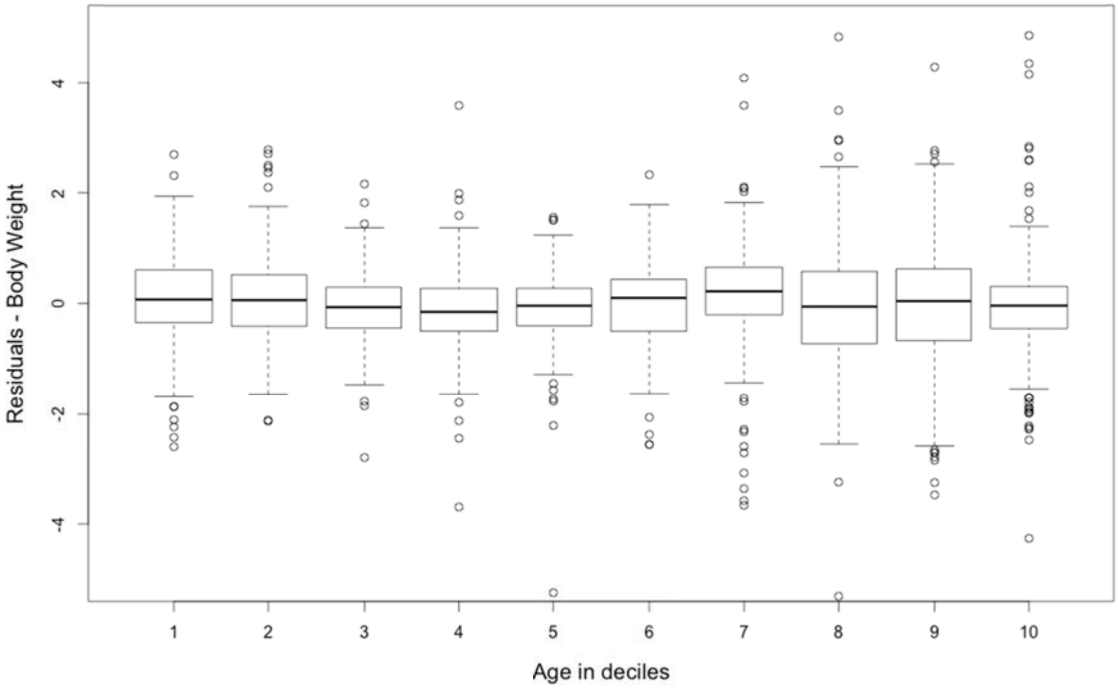

Residuals - Body Weight
4
2
0
-2
-4
1
2
3
4
5
6
7
8
9
10
Age in deciles

c.

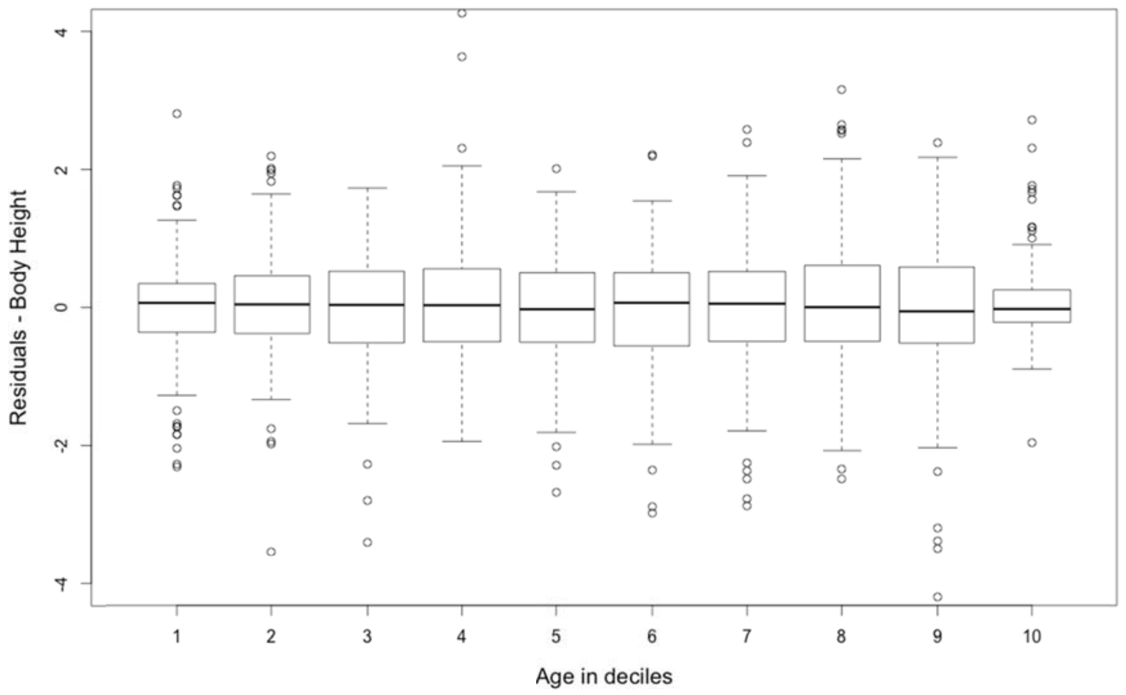

Supplementary Figure 2: Differences between observations and predictions from the model for height and weight (residual Standard Deviation). When the mean growth trajectory is of interest, the residuals in the model should have no trend over time, i.e. they should be centred on zero whatever the age. An assumption of homoscedasticity (constant variance over time) is required to estimate unbiased variances for statistical tests, which has little importance here. As can be seen from the graphs, the models estimate the mean growth trajectory with no systematic bias, for both periods A and B. The resulting models can be used to illustrate trajectories and to calculate growth characteristics and velocity curve.

a. Weight for the cohort A ($N_{individuals}$= 481, $N_{observations}$= 3089)

b. Weight for the cohort B ($N_{\mathbf{individuals}}$= 554, $\mathbf{N_{observations}= 1757}$)

c. Height for the cohort B ($N_{\mathbf{individuals}}$= 549, $\mathbf{N_{observations}= 1731}$)

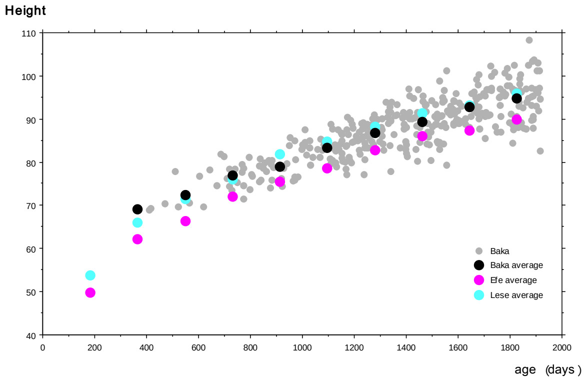

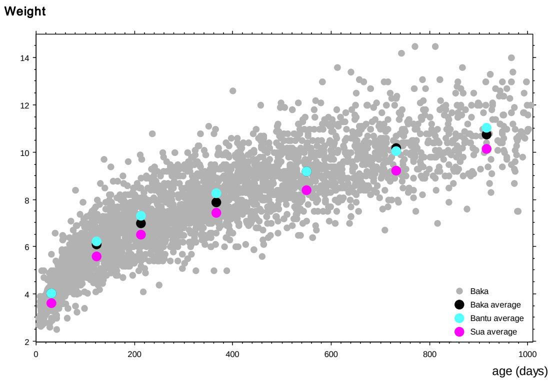

Supplementary Figure 3: Comparison between Baka and pygmies from East Africa. Baka average values are close to average non-pygmy's values (Lese, Bantu), whereas they are higher than those from Efe and Sua revealing two different growths processes responsible of pygmy phenotype.

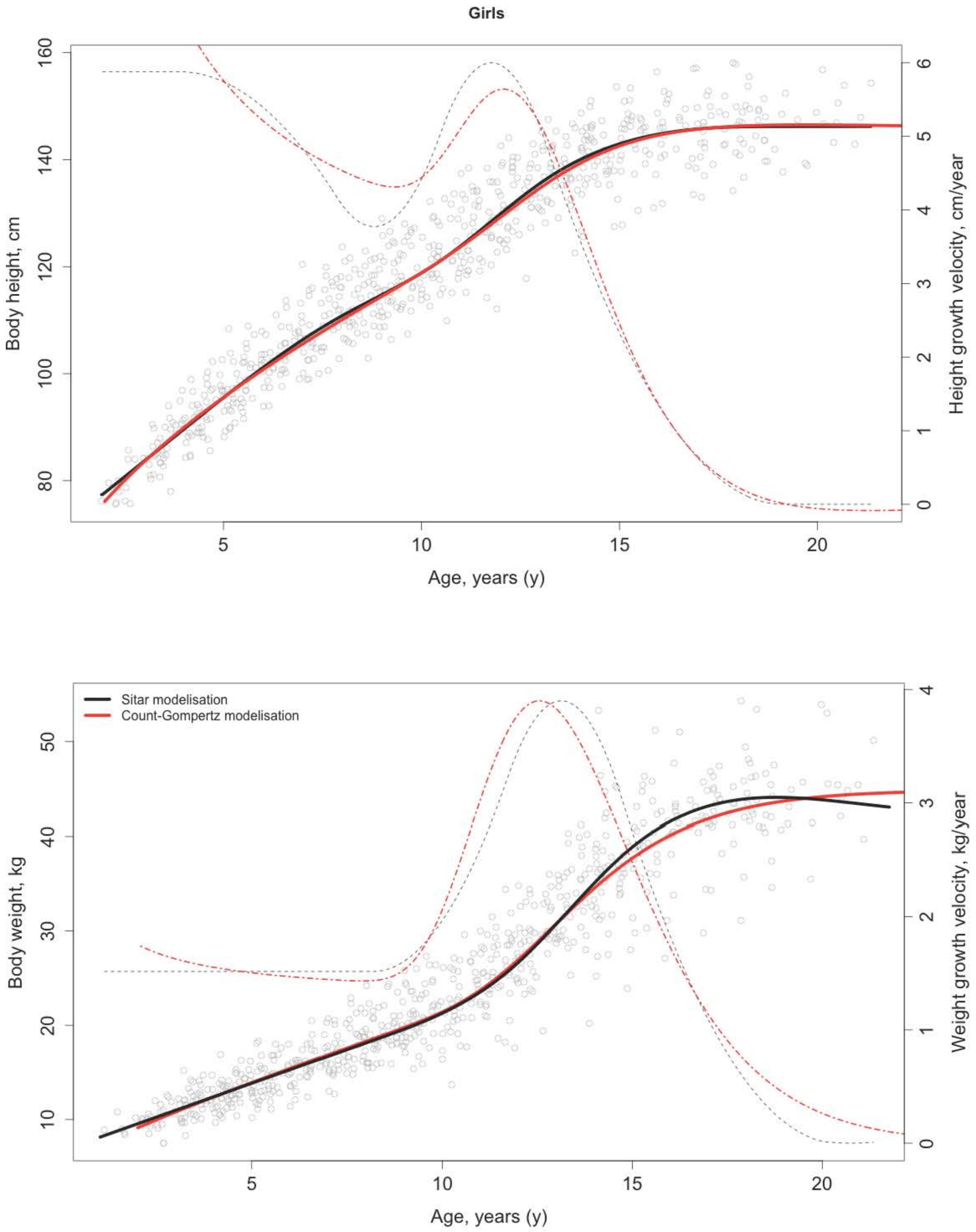

Supplementary Figure 4: Comparison of growth curved as fitted by the Count-Gompertz model and by SITAR model. Both models give very similar results and interpretation does not change using one or the other.

Supplementary Table 1: Age at Menarche in Sub-Saharan populations

	country	ethnicity/region	average	sd	CI (95%)	median	sd	CI (95%)
This study	Cameroon	Baka				14.5		14.1 - 15.0
1	Cameroon	Yaoundé<br>suburban<br>rural				13.18<br>13.98<br>14.27	1.08<br>1.55<br>1.65	
2	Congo		13.88 - 13.94§					
3	Dem.Rep.Congo	Bunya	15.67			14 - 15		
4	Dem.Rep.Congo	Kinshasa	13.61-13.99§			13.3		
5	Benin	Benin city	13.44	1.32	13.4-13.5			
6	Nigeria	Ilorin	13.21	1.29				
7	Nigeria	Enugu	13.3	1.09				
8	Nigeria	Port Harcourt	13.43	2.19				
9	Nigeria	Ife	13.94	1.31				
10	Nigeria	Zaria	14.2					
11	Nigeria	Ibadan	13.26-13.76 §					
12	Nigeria		13.53					
12	Zimbabwe		14.24					
13	Senegal					15.9		15.7 - 18.9
14	Senegal		16.1	2				
15	Ethiopia	Sawla	13.9	1.2	13.8 - 14	13 - 14		
16	Ethiopia	Amhara	13.72	1.31		14		
17	Tanzania		14.3	1.1				
18	South Africa	Transkei	15.02 - 15.42§			14 - 15		

§ Mean varies after the socioeconomic status.

Supplementary Table 2: Comparison of somatic growth (height & weight) between the Baka and other non-pygmies African groups

Height

Boys

Age	Baka°			Lunda & Tchokwe[19]			Somali[20]			Datoga[21]			Baganda[22]			Baka vs	Baka vs	Baka vs	Baka vs
years	X	N	SD	X	N	SD	X	N	SD	X	N	SD	X	N	SD	L&Tch	Somali	Datoga	Baganda
1.5	74.9	11	4.7				77.4	39	5.8	76.8	11	5.0	77.4	123	4.0		0.1123	0.2177	0.1123
2.5	80.3	27	3.9				84.2	45	6.5	84.7	16	4.7	87.1	91	4.6		***	***	***
3.5	86.6	56	4.2	86.8	1042	5.38	93.2	60	9.4	92.7	22	6.1	96.4	63	4.7	0.8049	***	***	***
4.5	91.9	62	4.6	93.5	742	6.01	104.0	16	5.9	99.1	10	5.7	105.4	202	5.7	**	***	***	***
5.5	97.4	66	5.3	99.1	856	6.04	109.5	12	4.3	104.0	14	8.9	109.8	175	5.1	**	***	***	***
6.5	101.4	66	5.8	104.8	840	6.69	115.9	22	9.8	112.4	12	5.9	112.0	110	4.2	***	***	***	***
7.5	106.1	74	6.1	109.0	747	7.44	120.4	57	7.1	118.2	9	9.6				***	***	***	
8.5	111.1	70	5.5	112.8	792	7.16	126.3	60	5.9	124.2	5	3.9				*	***	***	
9.5	115.6	62	5.1	117.9	774	8.45	130.4	49	6.7	126.3	12	2.9				***	***	***	
10.5	119.6	49	6.3	122.1	737	8.85	135.0	51	5.6	132.2	9	9.4				**	***	***	
11.5	122.8	47	5.7	126.6	835	8.27	138.5	59	6.5	136.1	10	5.8				***	***	***	
12.5	127.2	53	5.5	132.5	826	8.91	142.8	67	6.6	142.6	9	9.2				***	***	***	
13.5	131.9	40	5.9	137.9	837	9.10	150.0	67	7.2	139.0	5	8.7				***	***	***	
14.5	138.4	33	7.5	145.8	1179	9.35	155.2	59	10.6	141.4	8	9.4				***	***	*	
15.5	141.7	26	7.4	152.3	512	9.42	164.3	62	7.6	145.4	3	2.6				***	***	*	
16.5	145.5	20	8	155.9	542	6.77	169.3	53	6.1	159.1	2	2.8				***	***	***	
17.5	150.4	23	5.8	157.7	547	7.01	171.6	37	4.4							***	***		
18.5	152.8	11	4.6	158.3	582	7.13	170.9	36	6.8							**	***		
19.5	155.4	12	4.9	159.7	453	7.23										*			

Girls

Age	Baka°			Lunda & Tchokwe[19]			Somali[20]			Datoga[21]			Baganda[22]			Baka vs	Baka vs	Baka vs	Baka vs
years	X	N	SD	X	N	SD	X	N	SD	X	N	SD	X	N	SD	L&Tch	Somali	Datoga	Baganda
1.5	73.3	6	3.5				74.1	42	6.4	76.9	19	5.2	76.5	91	4.4		0.6105	0.0556	0.0785
2.5	79.3	27	3.5				83.7	51	7.0	89.5	17	7.0	86.1	77	4.5		***	***	***
3.5	86.2	66	4.4	86.7	1024	5.56	92.0	44	5.9	94.6	20	9.9	94.5	50	4.7	0.3322	***	***	***
4.5	92.6	64	4.7	93.3	790	6.00	103.8	18	3.6	97.5	19	8.6	105.5	205	5.5	0.2153	***	***	***
5.5	98.2	63	5.1	99.4	892	6.36	109.9	10	3.7	107.6	24	9.7	110.4	213	4.8	0.0692	***	***	***
6.5	104.6	43	5.7	105.1	805	6.68	117.4	37	5.7	111.5	10	8.1	113.6	97	4.6	0.5929	***	***	***
7.5	108.7	55	5.8	109.8	800	7.22	119.7	47	5.0	120.2	14	6.2				0.1591	***	***	
8.5	113.7	53	5.4	113.5	892	7.60	125.6	65	5.6	125.7	6	5.1				0.7934	***	***	
9.5	118.0	55	6.1	118.6	821	8.47	131.4	53	5.9	125.1	16	8.2				0.4473	***	***	
10.5	121.9	44	5.9	122.8	698	9.06	138.5	61	6.9	131.9	12	10.2				0.3207	***	***	
11.5	127.6	54	6.6	128.0	767	8.60	142.9	31	5.7	135.1	12	7.0				0.6491	***	***	
12.5	133.3	54	6	133.7	741	8.77	147.9	53	6.8	137.4	12	7.1				0.6359	***	***	
13.5	137.3	49	5.5	138.8	645	8.46	153.9	52	5.9	149.2	7	10.6				0.052	***	***	
14.5	140.5	48	6.2	144.0	561	7.55	158.3	35	4.2	149.1	10	6.3				***	***	***	

15.5	143.5	39	6	146.9	498	7.61	159.0	62	5.2	152.4	9	8.1		***	***	***	
16.5	145.3	28	6.2	148.7	363	6.39	159.3	42	4.8	156.7	4	5.8		**	***	***	
17.5	145.2	27	6.6	149.8	319	7.01	158.5	25	6.3	166.8	2	5.6		**	***	***	
18.5	145.5	25	4.8	150.1	395	6.37	163.2	28	4.7	149.3	2	6.7		***	***	***	
19.5	145.6	10	5.3	150.3	444	6.59								*			

Weight

Boys

Age	Baka °			Somali[20]			Datoga[21]			Baganda[22]			Baka vs	Baka vs	Baka vs
years	X	N	SD	X	N	SD	X	N	SD	X	N	SD	Somali	Datoga	Baganda
1.5	9.28	563	1.24	9.3	86	1.8	9.8	11	1.1	10.62	457	1.18	0.7274	***	***
2.5	10.87	179	1.35	11.2	91	1.8	10.9	16	1.4	12.60	311	1.20	**	0.8038	***
3.5	11.79	72	1.2	12.8	93	2.1	12.8	22	2.5	14.66	165	1.02	***	***	***
4.5	12.93	63	1.44	15.3	36	3.6	15.6	10	2.6	17.33	210	2.28	***	***	***
5.5	14.34	66	1.8	17.2	17	2.5	15.7	14	2.3	18.31	200	2.13	***	***	***
6.5	15.45	66	2.02	19.1	22	3.0	17.6	12	2.7	18.63	128	1.64	***	***	***
7.5	16.86	74	2.48	21.3	57	3.3	20.2	9	4.0				***	***	
8.5	18.27	70	2.6	23.8	60	4.1	21.6	5	1.9				***	***	
9.5	20.23	62	2.92	25.2	49	4.1	23.2	12	2.0				***	***	
10.5	22.11	49	3.37	27.3	51	5.0	24.5	9	3.8				***	***	
11.5	23.89	47	3.32	29.9	59	5.3	27.6	10	2.5				***	***	
12.5	25.63	53	3.28	31.6	67	5.5	29.1	9	4.3				***	***	
13.5	28.66	39	4.43	36.0	67	7.3	29.7	5	5.0				***	0.1501	
14.5	33.38	33	5.64	38.8	59	7.7	30.1	8	4.8				***	***B	
15.5	35.77	26	6.2	44.4	62	6.0	35.1	3	2.7				***	0.5776	
16.5	39.41	20	7.78	49.6	53	6.1	36.5	2	5.9				***	0.124	
17.5	43.17	23	9.08	50.9	37	6.2							***		
18.5	46.73	11	8.79	50.3	36	8.1							***		

Girls

Age	Baka °			Somali[20]			Datoga[21]			Baganda[22]			Baka vs	Baka vs	Baka vs
years	X	N	SD	X	N	SD	X	N	SD	X	N	SD	Somali	Datoga	Baganda
1.5	8.82	514	1.24	9.0	89	1.7	8.4	19	1.4	10.20	275	1.03	***	***B	***
2.5	10.38	193	1.24	10.7	99	1.9	11.2	17	1.9	12.01	250	1.12	***	***	***
3.5	11.65	85	1.74	12.3	70	1.8	13.1	20	2.4	14.14	17	0.98	***	***	***
4.5	12.95	65	1.65	15.0	38	2.4	13.8	19	2.3	16.63	234	2.12	***	**	***
5.5	14.47	63	2.02	17.3	15	2.5	15.9	24	3.2	18.08	238	2.00	***	***	***
6.5	16.13	43	2.18	20.3	37	3.0	16.8	10	3.2	18.87	119	2.01	***	0.0516	***
7.5	17.46	55	2.39	20.7	47	2.9	19.9	14	2.4				***	***	
8.5	19.31	53	2.34	23.4	65	3.7	22.1	6	1.3				***	***	
9.5	21.23	55	2.9	26.9	53	4.2	21.5	16	3.9				***	0.4916	

10.5	22.71	44	3.4	29.2	61	4.5	23.7	12	4.2		***	0.0607	
11.5	26.04	54	3.53	33.1	31	6.6	27.0	12	4.2		***	0.0515	
12.5	29.50	54	3.53	34.6	53	6.0	28.6	12	6.6		***	0.0651	
13.5	32.87	49	4.42	41.5	52	8.1	34.2	7	6.5		***	0.0411	
14.5	36.27	48	5.73	45.9	35	7.7	35.5	10	5.3		***	0.3576	
15.5	38.63	39	5.1	48.4	62	8.2	38.0	9	8.2		***	0.4483	
16.5	41.24	27	4.2	50.4	42	8.6	38.3	4	7.4		***	**B	
17.5	42.85	27	4.63	49.0	25	8.0	46.2	2	1.4		***	***	
18.5	43.28	25	4.02	50.2	28	6.6	41.0	2	9.2		***	**B	

° this study

B = Baka values are higher.

*** = P < 0.001, ** = P < 0.01, * = P < 0.05 (*t*-test one sample).

Supplementary Table 3: Comparison of somatic growth (height & weight) between the Baka and non-pygmies African groups (girls and boys together)

Height

Age in years	Baka° X	N	SD	Keneba[23] X	N	SD	Baka vs Keneba
1.5	72.5	7	3.4	73.6	3.86	65	0.359
2	77.0	19	2.9	78.6	3.56	50	0.383
2.5	79.0	28	3.2	82.0	3.76	38	**
3	83.4	46	3.1	87.0	3.48	43	***
3.5	86.8	60	3.8	89.8	3.84	44	***
4	89.4	69	4.4	94.2	3.56	41	***
4.5	92.8	60	4	97.5	3.51	44	***
5	94.9	66	5.2	100.3	3.38	34	***

Weight

Age in years	Baka° X	N	S.D.	Keneba[23] X	N	S.D.	Baka vs Keneba
0.0	3	2		2.8	68	0.39	
0.25	5.47	342	0.84	5.4	81	0.78	0.1072
0.5	6.71	305	0.98	6.5	81	0.98	*** B
0.75	7.4	255	1.03	6.9	75	1.02	*** B
1	8.04	256	1.02	7.2	75	1.16	*** B
1.25	8.67	218	1.05	7.6	70	1.22	*** B
1.5	9.15	187	1.18	8.1	66	1.18	*** B
1.75	9.68	140	1.27	9.0	58	1.32	*** B
2	10.09	108	1.27	9.6	52	1.50	*** B
2.25	10.32	79	1.21	10.1	42	1.62	0.1084
2.5	10.73	51	1.23	10.8	38	1.32	0.6846
2.75	10.98	42	1.44	11.3	40	1.48	0.1591
3	11.29	33	1.27	11.9	41	1.37	**
3.5	11.63	31	1.3	12.7	38	1.31	***
4	12.33	26	1.39	13.9	38	1.57	***
4.5	12.98	18	1.25	14.8	37	1.56	***
5	13.48	24	1.52	15.6	29	1.43	***

° this study

B = Baka values are higher.

*** = P < 0.001, ** = P < 0.01, * = P < 0.05 (*t*-test one sample).

Supplementary Table 4: Comparison of somatic growth (height & weight) between the Baka and non-pygmies African groups

Height

Age in years	Girls: Baka° X	N	SD	Ime[24] X	N	SD	Baka vs Ime	Boys: Baka° X	N	SD	Ime[24] X	N	SD	Baka vs Ime
1								69.1	2	0.3	72.1	na	na	0.421
1.5	70.3	3	0.5	76.7	na	na	***	74.2	4	3.9	77.7	na	na	0.1631
2	76.8	6	1.8	82.8	na	na	***	77.1	13	3.3	82.5	na	na	***
2.5	78.7	19	3.4	85.6	na	na	***	79.8	9	2.7	86.1	na	na	***
3	83.2	20	3.4	89.1	na	na	***	83.6	26	2.8	89.9	na	na	***
3.5	86.3	39	3.9	92.7	na	na	***	87.7	21	3.3	93.2	na	na	***
4	89.8	32	4.4	95.5	na	na	***	88.9	34	4.4	96.7	na	na	***
4.5	93.1	34	4.1	98.8	na	na	***	92.5	26	4	99.0	na	na	***
5	96.2	32	4.8	101.3	na	na	***	93.2	34	5.4	102.1	na	na	***

Weight

Age in years	Girls: Baka° X	N	SD	Ime[24] X	N	SD	Baka vs Ime	Boys: Baka° X	N	SD	Ime[24] X	N	SD	Baka vs Ime
0.25	5.28	265	0.87	5.08	na	na	***B	5.62	271	0.93	5.36	na	na	***B
0.5	6.46	209	0.92	6.26	na	na	***B	6.93	238	0.99	6.58	na	na	***B
0.75	7.20	194	1.04	6.85	na	na	***B	7.61	195	1.06	7.53	na	na	0.3128
1	8.01	243	1.1	7.89	na	na	0.0939	8.47	312	1.01	8.21	na	na	***B
1.5	8.87	268	1.16	9.25	na	na	***	9.47	264	1.15	9.53	na	na	0.4023
2	9.89	164	1.12	10.16	na	na	***	10.21	165	1.37	10.70	na	na	***
2.5	10.44	99	1.27	11.34	na	na	***	11.01	86	1.21	11.61	na	na	***
3	11.07	44	1.34	12.52	na	na	***	11.28	53	1.29	12.66	na	na	***
3.5	11.80	51	1.87	13.20	na	na	***	11.89	29	1.14	13.65	na	na	***
4	12.10	33	1.62	13.88	na	na	***	12.36	38	1.28	14.06	na	na	***
4.5	13.06	34	1.38	14.74	na	na	***	13.16	26	1.31	14.83	na	na	***
5	14.17	32	2.06	15.38	na	na	***	13.07	35	1.71	15.92	na	na	***

° this study
B = Baka values are higher.
*** = P < 0.001, ** = P < 0.01, * = P < 0.05 (*t*-test one sample).

Supplementary References

1. Pasquet, P. et al. Age at menarche and urbanization in Cameroon: current status and secular trends. *Ann. Hum. Biol.***26**, 89-97 (1999).

2. Lounana J. et al. Influence de la pratique sportive intensive et précoce sur l'âge à la ménarchedansune population de femmes congolaises. *Science & Sports***17**, 17-25 (2002).

3. Ekisawa, N., D'Hulst, C., Ghesquière, J. L'âge moyenne des premières règles chez les filles scolarisées de Bunia. *Bull. Et Mém. Soc. Anthropol. Paris* XIV° Série, **3**, 27-35 (1986).

4. Rashid-Tozin, R., Mugeni, I., Shango, L.,Omanga, U. Age moyen du menarche de la fille zaïroise à Kinshasa, Zaïre. *Ann. Soc. Belge Méd. Trop*. **64**, 69-74 (1984).

5. Onyiriuka, A. N., Abiodun, P. O., Onyiriuka, R. C., Ehirim, F. A.,Onyiriuka, E. P. A. Menarcheal age of nigerian urban secondary school girls in Benin City. *Reproductive Sys.Sexual Disord*.**1**, 106. (2012) doi:10.4172/2161-038X.1000106

6. Adebara, I. O.,Ijaiya, M. A. Relationship of biosocial factors and hemoglobin genotype with age of menarche among school girls in Ilorin, Nigeria. *Nepal J. Obst. Gyn.***7**, 19-24 (2012).

7. Modebe O. The effect of homozygous sickle cell disease onthe age at menarche in Nigerian school girls. *Ann. Hum. Biol.***14**, 181-185 (1987).

8. Ofuya, Z. M. The age at menarche in Nigerian adolescents fromtwo different socio-economic classes.*Online J. Health Allied Scs*. **4**, 3 (2007).

9. Abioye-Kuteyi, E. A. et al. Theinfluence of socieconomic and nutritional status on menarche in Nigerian schoolgirls. *Nutr. Health*.**11**, 185-95 (1997).

10. Anekwe, S. C., Haggai, D. P.,Ameh, N. in *Health sector reforms and maternal health* (eds. Omigbodun, A. O., Olayemi, O., Oladokun, A., Ladipo, O. A.) 297-299, SOGON (2006).

11. Oduntan, S. O., Ayeni, O., Kale, O. O. The age of menarche in Nigeria girls. *Ann. Hum. Biol*. **3**, 269-274 (1976).

12.Akinboye, J. O. Secondary sexual characteristics and normal puberty in Nigerian and Zimbabwean adolescents. *Adolescence* **19**, 483-492 (1984).

13. Garnier, D., Simondon, K.,Bénéfice, E. Longitudinal estimates of puberty timing in Senegalese adolescent girls. *Am. J. Hum. Biol*. **17**, 718-730 (2005).

14. Simondon, K. B., Simon, I.,Simondon, F. Nutritional status and age at menarche of Senegalese adolescents. *Ann. Hum. Biol*. **24**, 521-532 (1997).

15. Ayele, E., Berhan, Y. Age at menarche among in-school adolescents in Sawla town, South Ethiopia.Ethiop. *J. Health Sci*. **23**, 189-200 (2013).

16. Abera Y. Menarche, Menstruation related Problems and Practices among adolescent high school girls in Addis Ababa, 2003/04. Ph D Thesis, University Addis Ababa (2004).

17. Re¸bacz, E. Age at menarche in schoolgirls from Tanzania in light of socioeconomic and sociodemographic conditioning. *Coll. Antropol*. **33**, 23–29 (2009).

18. Burrell, R. J. W., Healy M. J. R., Tanner J. M. Age at menarche in South African Bantu schoolgirls living in the Transkei reserve. *Hum. Biol.***88**, 250-261 (1961).

19. Santos David, J. H. Height growth of melanodermic natives in Northeastern Lunda (Angola). *S. Af. J. med. Sci*. **37,** 49-60 (1972).

20. Gallo, P. G., Mestriner, M. F. Growth of children in Somalia. *Hum. Biol*. **52**, 547-561 (1980).

21. Sellen, D. W. Growth pattern among seminomadic pastoralists (Datoga) of Tanzania. *Am. J. Phys. Anthopol*. **109**, 187-209 (1990).

22. Rutishauser, I. H. Heights and weights of middle class Baganda children. *The Lancet* **412**, 565-567 (1965).

23. McGregor, I. A., Rahman, A. K., Thompson, B.The growth of young children in a Gambian village.Trans. Roy. Soc. Tropical Medecine & Hygiene 62, 341-352 (1968).

24. Morley D.C., Woodland M., Martin W.J., Allen J. Heights and weights of West African village children from birth to the age of five. *West African Medical Journal* 17, 8–13 (1968).
